# FEV_6_ as screening tool in diagnosis of obstructive airway disease

**DOI:** 10.4103/0970-2113.71976

**Published:** 2010

**Authors:** Sourabh Aggarwal

**Affiliations:** *University College of Medical Sciences, New Delhi, India. E-mail: drsourabh79@gmail.com*

Sir,

First of all, I would like to congratulate the editorial team of Lung India on getting this journal indexed with Pubmed.[1] However, the journey has just begun and I would like to wish them all the best for bright future.

I read the article ‘FEV_6_ as screening tool in spirometric diagnosis of obstructive airway disease’[2] by Malolan A *et al*. with interest. The authors have done a commendable effort to study such a topic in Indian setup and their efforts should be appreciated. I would like to share my views to the same.

The result of the study needs to be supplemented with a large multi-centered study before results can be generalized. Also, the availability of the necessary facilities to carry out such screening is not available in most parts of India and lot needs to be done before it can be implemented at a larger level. Nonetheless, authors have done a great job and needs to be applauded.
